# Genetic Diversity, Virulence, Race Profiling, and Comparative Genomic Analysis of the *Fusarium oxysporum* f. sp. *conglutinans* Strains Infecting Cabbages in China

**DOI:** 10.3389/fmicb.2019.01373

**Published:** 2019-06-25

**Authors:** Xing Liu, Miaomiao Xing, Congcong Kong, Zhiyuan Fang, Limei Yang, Yangyong Zhang, Yong Wang, Jian Ling, Yuhong Yang, Honghao Lv

**Affiliations:** Institute of Vegetables and Flowers, Chinese Academy of Agricultural Sciences, Key Laboratory of Biology and Genetic Improvement of Horticultural Crops, Ministry of Agriculture, Beijing, China

**Keywords:** cabbage fusarium wilt, *Fusarium oxysporum* f. sp. *conglutinans*, genetic diversity, virulence, molecular characterization, genomic comparisons

## Abstract

Cabbage Fusarium wilt (CFW) caused by *Fusarium oxysporum* f. sp. *conglutinans* (FOC) is known to significantly affect yield and quality of cabbages worldwide. CFW was first detected in New York, NY, United States, and has now spread to almost all cabbage-planting areas, including a recent outbreak of the disease in China. However, it was unknown whether the FOC strains emerged in China differed from the strains in other areas of the world. From 2009 to 2018, we collected Chinese FOC isolates and compared them to the races 1 and 2 strains in other areas to define their characteristics. Race tests indicated that most of the Chinese FOC strains belonged to race 1 and were more virulent than type strain 52557. To evaluate the genome level diversity, we performed next-generation sequencing and genome assembly for the race 2 strain 58385. Based on the assembled genome, we discovered abundant single-nucleotide polymorphisms and 645 insertion–deletions (InDels) compared with the race 1 strain FGL03-6 by comparative genomic analysis and showed that all FOC race 1 strains have a low genetic variability, with a genomic background distinct from 58385. Furthermore, the internal transcribed spacer, elongation factor-1α, and whole-genome InDel variation studies suggested that the last might be a powerful tool in phylogenetic as well as evolution analysis for *F. oxysporum* Schlechtend.: Fr. The race, virulence, and genome-based variation profiles could contribute to our knowledge of FOC diversity and support the studies of pathogen characterization in genomic era and also provide clues for CFW-resistance breeding. To our knowledge, this is the first extensive survey conducted for FOC strains.

## Introduction

*Fusarium oxysporum* Schlechtend.: Fr. (FO) is a widespread soil-borne fungal species which includes pathogenic and nonpathogenic strains. The pathogenic strains can cause wilt or root rot in more than 120 plant species, among which many are economically important horticultural and agricultural crops, such as tomato, melon, banana, and cotton (Gordon, 1965; Appel and Gordon, 1996). Based on their host specificity, the pathogenic strains of *F. oxysporum* are divided into *formae speciales* (f. sp.), some of which can be further divided into physiological races (Gordon and Martyn, 1997; Agrios, 2005; Berrocal-Lobo and Molina, 2008).

*Fusarium oxysporum* f. sp. *conglutinans* (Wollenw.) Snyder & Hansen (FOC), the causal agent of cabbage Fusarium wilt (CFW), is a worldwide threat to cabbage production, resulting in severe economic losses (Booth, 1971). In the early stage of CFW, the veins of the lower leaves appear reticular yellowing, then the symptoms spread from bottom to top, and finally the whole plant wilts and dies. It is clearly visible that the vascular tissue of the shortening stem turns brown in cross-sections (Walker and Hooker, 1945; Pietro et al., 2010). FOC can survive for decades in the soil even without any host, making traditional physical or chemical control difficult. Although resistant (R) cultivars of cabbage can be used to control the disease, the appearance of new pathogenic races usually overcomes host resistance (Park et al., 2002). FOC race 1 is the most prevalent race and has been found worldwide (Walker and Blank, 1934; Ramirez-Villupadua et al., 1985), while race 2 has been reported only in the United States and Russia until now and can overcome type-A resistance pattern inherited by single gene (Bosland and Williams, 1988; Morrison et al., 1994).

The cabbage is an important vegetable crop, a source of rich nutrition, has a strong adaptability, and is widely cultivated in China, with an annual planting area of about 900,000 ha (Yang et al., 2016), accounting for approximately 30% of the world production^1^. In 2001, CFW was first discovered in Yanqing County, Beijing, China, and the damage area has been increasing since then, threatening approximately one-third of the summer and autumn cabbage-growing areas in Northern China (Li et al., 2003; Lv et al., 2011, 2014b). Only a few isolates have been collected and subjected to pathogenicity and race tests, and their molecular characteristics are largely unknown, restricting our understanding of this pathogen and hindering the process of R cabbage variety breeding and control of CFW.

In the case of parasitism, the plant and pathogen have interacting and constantly selecting genetic changes. Pathogen evolution may result in change in virulence, which in turn results in new or different races that could overcome plant defense mechanisms. Typically, the arms race is between tomato and the wilt pathogen *F. oxysporum* f. sp. *lycopersici* (Sacc.) Snyder & Hansen (FOL): Bohn and Tucker (1939) firstly identified the *I* gene in wild tomato [*Lycopersicon pimpinellifolium* (L.) Mill.], which gives resistance to FOL race 1. But FOL race 2 emerged rapidly worldwide and became virulent on tomato varieties carrying the *I* gene. Subsequently, the *I-2* gene was identified in the 1960s and proved to be effective in controlling FOL race 2. Twenty years later, FOL race 3 was isolated from wilted tomato varieties carrying the *I-2* gene (Volin and Jones, 1982). More recently, two R genes (*I-3* and *I-7*) against FOL race 3 have been identified in *Lycopersicon pennellii* (Corr.) D’Arcy (Lim et al., 2006; Catanzariti et al., 2015; Gonzalezcendales et al., 2016). However, it is unknown when the new race will emerge. Therefore, it is important to monitor the virulence and race dynamics of pathogens and formulate appropriate breeding strategies for disease management.

Fast and accurate identification of pathogen isolates is necessary for disease management and control. Traditional research on pathogen identification and genetic variation mainly relies on morphology observation and limited molecular methods (Bosland and Williams, 1987; Kistler et al., 1987, 1991). In the genomic era, as increasingly more reference genomes for plant pathogens are made public, several rapid and reliable molecular methods to detect and identify plant pathogens are now available (Jurado et al., 2006). For instance, the application of Loop-mediated isothermal AMPlification (LAMP)-based assays allows us to identify a broad range of plant pathogens by offering a rapid, accurate, and cost-effective diagnostic tool (Ward and Harper, 2012; Sillo et al., 2018; Zhang et al., 2019). Some molecular methods of identification are based on DNA polymorphisms, such as random-amplified polymorphic DNA (RAPD), amplified fragment length polymorphism (AFLP), insertion–deletion (InDel), simple sequence repeat (SSR), and single-nucleotide polymorphism (SNP); others are based on specific gene sequences, e.g., internal transcribed spacer (ITS) of ribosomal DNA (rDNA), elongation factor-1α (EF-1α), β-tubulin, and calmodulin gene (Baayen et al., 2000; Skovgaard et al., 2001). These methods have been successfully applied in identification and variation studies of various Fusarium wilt pathogens (Haegi et al., 2013; Umesha et al., 2015; Kashiwa et al., 2016).

Due to the fact that research on FOC isolation and identification has not been carried out systematically in China, it is unclear whether the Chinese FOC isolates differ from other worldwide FOC strain collections, especially in terms of molecular characteristics. Thus, in the current study, we collected representative isolates from the most affected regions in China and performed pathogenicity and race tests. Genetic diversity analysis based on ITS sequences and EF-1α gene sequences was conducted for them. We also performed whole-genome re-sequencing of race 2 strain 58385 for comparative genomic analysis. Based on these results, the whole-genome variations were analyzed, and comparisons with other FOC strains, as well as with other *formae speciales*, were conducted. These results could contribute to the knowledge of the population structure, genomic diversity, virulence, and race dynamics of FOC strains and promote the development of CFW disease-management strategies.

## Materials and Methods

### Pathogen Isolates

From 2009 to 2018, surveys were conducted in cabbage-growing areas of China. Twelve regions were selected to collect diseased plant samples in six CFW infested provinces of China. In these regions, one to four diseased fields were selected to collect diseased samples. Pathogens were isolated from diseased plant samplings showing typical CFW symptoms, including stunting, yellowing, wilting, or even the death of whole plants. The protocol of pathogen isolation and pathogenicity tests was performed as previously described (Lv et al., 2011; Liu et al., 2017b). Diseased plant leaf samples (showing symptoms) were disinfected and cultured on potato dextrose agar (PDA) until the hyphae had grown. Single-spore colonies of all isolates were obtained for subsequent tests (Table 1). To confirm whether the pathogenic isolates were FOC, the morphology characteristics of the colonies, hyphae, microconidia, and macroconidia, as well as race test, were performed (see the protocol below), and the molecular characteristics of the ITS and EF-1α gene sequences was analyzed (see the protocol below).

**TABLE 1:** Accession numbers, host, and collection area of FOC and other *Fusarium oxysporum* isolates used in this study.

**Isolate No.**	**Forma specialis**	**Host^a^**	**Year^b^**	**Origins^c^**
FGL03-6	*conglutinans*	Cabbage	2008	Yanqing, Beijing, China
FOYQ-2	*conglutinans*	Cabbage	2011	Shenjiaying, Yanqing, Beijing, China
FOYQ-3	*conglutinans*	Cabbage	2011	Kangzhuang, Yanqing, Beijing, China
FOYQ-4	*conglutinans*	Broccoli	2012	Kangzhuang, Yanqing, Beijing, China
FOSN	*conglutinans*	Cauliflower	2013	Haidian, Beijing, China
FOCP-1	*conglutinans*	Cabbage	2013	Nankou, Changping, Beijing, China
FOCP-2	*conglutinans*	Cabbage	2018	Nankou, Changping, Beijing, China
FOHL	*conglutinans*	Cabbage	2011	Huailai, Hebei Province, China
FOXT	*conglutinans*	Cabbage	2017	Xingtai, Hebei Province, China
FOLT	*conglutinans*	Cabbage	2018	Laoting, Hebei Province, China
FOSY-1	*conglutinans*	Cabbage	2009	Pingtou, Shouyang, Shanxi Province, China
FOSY-2	*conglutinans*	Cabbage	2011	Nanyanzhu, Shouyang, Shanxi Province, China
FOPT	*conglutinans*	Cabbage	2012	Pingtou, Shouyang, Shanxi Province, China
FOTY-1	*conglutinans*	Cabbage	2009	Taiyuan, Shanxi Province, China
FOTY-2	*conglutinans*	Cabbage	2011	Taiyuan, Shanxi Province, China
FOLZ-1	*conglutinans*	Cabbage	2012	Honggu, Lanzhou, Gansu Province, China
FOLZ-2	*conglutinans*	Cabbage	2013	Yuzhong, Lanzhou, Gansu Province, China
FODX	*conglutinans*	Cabbage	2012	Neiguan, Dingxi, Gansu Province, China
FOJY	*conglutinans*	Cabbage	2016	Jingyang, Xianyang, Shaanxi Province, China
FOCQ	*conglutinans*	Cabbage	2017	Longshi, Hechuan, Chongqing, China
52557	*conglutinans*	Cabbage	2012	WI, United States
58385	*conglutinans*	Cabbage	2012	CA, United States
CS20	*–*	Non-pathogenic	2009	United States
FOCAM	*conglutinans*	Cabbage	2009	United States
A8	*conglutinans*	Cabbage	–	Italy
FO-Tom	*lycopersici*	Tomato	–	China
FO-Cow	*phaseoli*	Cowpea	–	China
FO-Cuc	*cucumerinum*	Cucumber	–	China
FO-Pep	*capsicum*	Pepper	–	China
FO-Egg	*melongenae*	Eggplant	–	China

*${}^{a}$Plant species infected by the isolate. ${}^{b}$Year isolate was obtained. ${}^{c}$Location of the diseased field.*

The FOC or FO strains collected elsewhere in the world were also used in this study: (i) 52557 (ATCC^®^ 52557^TM^ , obtained from the American Type Culture Collection) and 58385 (ATCC^®^ 58385^TM^), the type strains for race 1 and race 2 of FOC, respectively, which were isolated from diseased cabbage plants in Wisconsin and California in the United States, respectively (Ramirez-Villupadua et al., 1985; Bosland and Williams, 1987); (ii) FOCAM, race 1 and a nonpathogenic FO strain CS20, provided by the US Department of Agriculture–Agricultural Research Service (USDA–ARS). Furthermore, another strain, A8, race 1 of FOC, was obtained from Italy. These strains were used in the comparison of pathogenicity, race, and genetic studies with the Chinese strains. Additionally, five other FO *formae speciales* (Table 1): *F. oxysporum* f. sp. *lycopersic*i (FO-Tom), *F. oxysporum* f. sp. *phaseoli* (FO-Cow), *F. oxysporum* f. sp. *cucumerinum* (FO-Cuc), *F. oxysporum* f. sp. *capsicum* (FO-Pep), and *F. oxysporum* f. sp. *melongenae* (FO-Egg) were provided by the Institute of Vegetables and Flowers, Chinese Academy of Agricultural Sciences (IVF–CAAS) and used in the genetic studies for ITS and EF-1α gene tests as well as in whole-genome InDel analysis.

### Race and Pathogenicity Experiments

The previously reported two cabbage accessions, i.e., “Golden Acre 84” and “Badger Inbred 16” (Table 2), provided by USDA–ARS were used as differential hosts to identify races and pathogenicity of the FOC isolates (Bosland and Williams, 1987). Three additional accessions, including “96-100,” “01-20,” and “Fast Vantage” (Table 2), were also used according to their resistance to different races in the pathogenicity and race tests in the previous studies (Lv et al., 2013; Liu et al., 2017a, b).

**TABLE 2:** Cabbage accessions and resistance used in this study for pathogenicity and race tests.

		**Resistance**	**Resistance**	
**Accession name**	**Accession type**	**to race 1**	**to race 2**	**References**
Badger Inbred 16	Cabbage inbred line	HR^a^	HR	(Bosland and Williams, 1987)
Golden Acre 84	Cabbage F_1_	HS^b^	HS	(Bosland and Williams, 1987)
96-100	Cabbage inbred line	HR	HS	(Lv et al., 2013)
01-20	Cabbage inbred line	HS	HS	(Liu et al., 2017a)
Fast Vantage	Cabbage F_1_	HS	HS	(Liu et al., 2017b)

*${}^{a}$Highly resistant to the race. ${}^{b}$Highly susceptible to the race.*

The root-dipping method was adopted according to our previous studies (Lv et al., 2013, 2014a). The cabbage seeds underwent accelerated germination in a 28°C incubator under an all-darkness condition. Then they were sown in plastic pots (9 cm × 9 cm × 9 cm) with sterilized substrate (soil:vermiculite:peat = 1:1:1) and cultivated in a greenhouse with a temperature of 28°C in the day and 20°C in the night until the third leaf stage. FOC strains were incubated in Erlenmeyer flasks containing complete medium (CM with casein acids hydrolysate 3 g/L and casein enzyme hydrolysate 3 g/L and yeast extract 6 g/L and sucrose 10 g/L) on a rotary shaker (200 rpm, 28°C) for 3 days. A conidial suspension was isolated with a gauze and diluted to 1 × 10^6^ conidia/mL using a hemocytometer. Roots of the seedlings were dipped in the conidial suspension for 15 min, and then they were re-planted in plastic pots with sterilized substrate and maintained in the greenhouse with the day temperature of 27–29°C and night temperature of 23–25°C.

Disease severity was measured 8 days after inoculation as described in our previous study (Liu et al., 2017b). Individual plants were rated for disease severity based on the following scale: 0 = no symptoms; 1 = one leaf yellowing slightly; 2 = one or two leaves yellowing moderately; 3 = half of the leaves yellowing seriously and/or wilting; 4 = all leaves except the heart leaves yellowing seriously or wilting; and 5 = all leaves yellowing, wilting seriously, or plant death. Eight days after inoculation, we calculated the disease index (DI) with the formula: DI = [Σ(disease rate × the number of plants at the corresponding rate)/(total number of the plants × the highest disease rate)] × 100. Based on the DI, the resistance level of the hosts was recorded as highly resistant (HR) (0 ≤ DI < 10), R (10 ≤ DI < 30), moderately resistant (MR) (30 ≤ DI < 50), susceptible (S) (50 ≤ DI < 70), and highly susceptible (HS) (DI ≥ 70) (Lv et al., 2011). Three replicates (nine plants for each replicate) were performed in the test. The race types were classified according to the host resistance levels described in Table 2.

### Molecular Characterization Based on ITS and EF-1α Gene Sequences

All FOC and FO isolates were cultivated on a PDA medium for 5 days at 28°C, and then approximately 500 mg of mycelium from each isolate was used for DNA extraction using the cetyl trimethyl ammonium bromide (CTAB) method (Möller et al., 1992). DNA samples were diluted to a concentration of approximately 50 ng/μL and stored at −20°C prior to use. The quality and quantity of DNA were estimated using a ND-1000 spectrophotometer (Thermo Fisher Scientific Inc., Wilmington, DE, United States).

Molecular analysis was performed including the ITS gene region and the translation EF-1α gene. The ITS region was amplified with primers ITS1 (5′-TCCGTAGGTGAACCTGCGG-3′) and ITS4 (5′-TCCTCCGCTTATTGATATGC-3′) (White et al., 1990), and the EF-1α gene was amplified with primers ef1 (5′-ATGGGTAAGGAAGACAAGAC-3′) and ef2 (5′-GGAA GTACCAGTGATCATGTT-3′) (O’Donnell et al., 1998a, b). PCR reactions were conducted in a 20 μL final volume. Each reaction was comprised of 2 μL of DNA template, 2 μL of 10× PCR buffer (Mg^2+^ included), 0.4 μL of Taq polymerase (2.5 U/μL), 1.6 μL of deoxynucleotide triphosphate (dNTPs) (2.5 mM each), 0.8 μL forward and reverse primers (10 μM), and 12.4 μL double-distilled H_2_O. The amplification reactions were conducted in a GeneAmp PCR system 9700 thermal cycle (Life Technologies Co., Carlsbad, CA, United States), using conditions specific for each target gene region. Conditions for the amplification of the ITS region and EF-1α gene were denaturation at 95°C for 3 min, 33 cycles of 30 s at 95°C, 30 s at 55°C, and 1 min at 72°C followed by a final extension time of 10 min at 72°C. The PCR products were purified and cloned into the pMDTM19-T Vector (TaKaRa Bio Inc., Kusatsu, Shiga, Japan) following the manufacturer’s protocol. The positive cloning was selected on Luria-Bertani agar containing ampicillin (100 mg/mL) and sequenced using the universal primers derived from the sequence flanking the vector.

Sequencing was performed using ABI 3730xl DNA Analyzers (Applied Biosystems, Foster City, CA, United States). The nucleotide sequences of the ITS region and EF-1α gene were aligned with ClustalW using the Molecular Evolutionary Genetics Analysis software package, version 6 (MEGA6) (Tamura et al., 2013). Phylogenetic tree was generated by the neighbor-joining (NJ) method from the alignment of the nucleotide sequences with MEGA6. Bootstrap analysis with 1000 replications was performed to assess group support. Branch length was proportional to the number of nucleotide changes (bar).

### Comparative Genomics Analysis of Race 1 and Race 2 and Other FO Strains

The DNA sample of race 2 strain 58385 was extracted and subjected to whole-genome resequencing. One microgram of genomic DNA was sequenced using the Illumina HiSeq X Ten platform (Illumina Inc., Beijing, China), specifying 150-bp paired-end reads with an insert size of 500 bp. To facilitate comparative genomics analysis, the 58385 genome was assembled using the softwares SOAPdenovo 2.04 and Gapcloser 1.12 (Luo et al., 2012). The genome of FOC strain 54008 was downloaded from National Center for Biotechnology Information (NCBI) (GenBank Accession No. LPZQ00000000) and used as the reference genome. The gene annotation was performed using AUGUSTUS version 3.2.2 (Stanke et al., 2004). Also, the genome data for FGL03-6 (Accession No. NRHZ00000000) were downloaded from NCBI and used for subsequent comparative analysis (Lv et al., 2018).

The FGL03-6 genome was used as the reference to scan for the SNPs and InDels of the 58385 genome. MUMer software (Kurtz et al., 2004) was used for whole-genome alignment and finding potential SNPs. The sequence 100 bp upstream and downstream of the SNP locus on the reference was retrieved using BLAT version 36 (Kent, 2002)^2^ and confirmed again with the 58385 genome, and only the SNPs with both sequence alignment length >100 bp and single alignment position were kept. Finally, reliable SNPs were acquired using BLAST (Altschul et al., 1990), RepeatMaske (Saha et al., 2008), and TRF (Benson, 1999) software to remove those located in the repeat genome region of FGL03-6. For InDel analysis, LASTZ software (Chiaromonte et al., 2002) and axtCorrection, axtSort, and axtBest programs (Harris, 2007) were first adopted to generate a primary InDel search result; then, 150 bp downstream and upstream of the InDel loci were retrieved and aligned with resequencing reads using BWA (Li and Durbin, 2009)^3^, and SAMtools (Li et al., 2009)^4^ to filter for reliable InDels. The SNPs and InDels between FGL03-6 and 58385 on the longest 100 scaffolds of FGL03-6 were presented using Circos version 0.69 (Krzywinski et al., 2009)^5^. GC content and GC-Skew value distribution were also analyzed and presented.

To obtain an evolutionary status of the FOC strains, the two genome assemblies were aligned with 25 strains from the 22 published FO *formae speciales* genomes, including *ciceris*, *conglutinans* strain 1, *conglutinans* race 2 54008, *cubense* race 1, *cubense* race 4, *cubense* tropical race 4, *cucumerinum*, *gladioli*, *lagenariae*, *lilii*, *luffae*, *lycopersici*, *medicaginis*, *melongenae*, *melonis*, *momordicae*, *narcissi*, *nicotianae*, *niveum*, *pisi*, *radicis-cucumerinum*, *radicis*-*lycopersici*, *raphanin*, *tulipae*, and *vasinfectum*, with GenBank Assembly Accession Nos. GCA_001757345.1, GCA_001519035.1, GCA_000260215.2, GCA_000350345.1, GCA_000350365.1, GCA_000260195.2, GCA_001702515.1, GCA_002233895.1, GCA_002233875.1, GCA_002234115.1, GCA_002233855.1, GCA_000149955.2, GCA_001652425.1, GCA_001888865.1, GCA_001703215.1, GCA_002233795.1, GCA_002233775.1, GCA_002234055.1, GCA_001702745.1, GCA_000260075.2, GCA_001702695.2, GCA_000260155.3, GCA_000260235.2, GCA_002233805.1, and GCA_000260175.2, respectively. The genome of *F. oxysporum* f. sp. *lycopersici* has the chromosome assembly level deposited in GenBank, and used as reference for SNPs analysis for the other 26 genomes. SNPs were selected according to previously described criteria. These SNPs for each of the 27 FO strains were joined together to produce FASTA format sequences with the same length. PhyML version 3.0 (Guindon et al., 2010) software was adopted to construct the phylogenetic tree using the maximum-likelihood method (Guindon and Gascuel, 2003), with a bootstrap value of 1000. A circular dendrogram was presented using iTOL software version 4.1.1 (Letunic and Bork, 2016)^6^.

### Genome-Wide InDel Diversity Analysis of the FO Strains

The clean data generated by resequencing were aligned with FOC strain 54008 as the reference, using BWA version 0.7.12 (Li and Durbin, 2009)^7^ and SAMtools version 0.1.18 (Li et al., 2009)^8^. The InDels were characterized using GATK version 3.2.2 (van der Auwera et al., 2013)^9^. The InDels were acquired using the protocol described above. For high-quality InDels, the main parameter settings were specifying allele depth ≥3, align quality value ≥20, and variation quality value ≥50.

For the InDel lengths ≥3 bp, the primers were designed to amplify the DNA segment 150 bp downstream and upstream of the genetic variation of the loci using Primer version 3.0 software (Untergasser et al., 2012)^10^. All primers were filtered to retain unique primers with only one amplicon on the whole genome using BLAST tools.

The InDel loci for all the isolates were analyzed using the following protocol: (i) Genomic DNA was extracted from the mycelium of each isolate using the CTAB method (see the protocol above), and samples were then diluted to approximately 50 ng/μL and used as templates; (ii) PCR reactions were conducted in a 20 μL reaction volume, comprised of 2 μL DNA template, 2 μL of 10× PCR buffer (Mg^2+^ included), 0.4 μL of Taq polymerase (2.5 U/μL), 1.6 μL of dNTPs (2.5 mM each), 0.8 μL of forward and reverse primers (10 μM), and 12.4 μL of double-distilled H_2_O. The amplification reactions for InDel loci were as follows: 95°C for 5 min and 35 cycles of 95°C for 30 s, 58°C for 30 s, and 72°C for 1 min; 72°C for 10 min; (iii) the protocol of polyacrylamide gel electrophoresis analysis used as previously described (Liu et al., 2017a).

DNA fingerprint data generated by InDel markers were converted into binary matrix. Comparison of each profile for each of the primers was based on the presence (1) vs. absence (0) of InDel amplimers that migrated to the same position in the gel. Binary matrices were analyzed by NTSYS-PC (version 2.0; Exeter Biological Software, Setauket, NY, United States). Jaccard’s coefficients were clustered to generate a dendrogram by using SHAN clustering program using the unweighted paired group method with arithmetic average analysis (UPGMA) (Rohlf, 1998).

### Data Analyses

The statistical analyses for cultural characteristics and disease severity data were performed using SPSS20.0 softwares (SPSS, Chicago, IL, United States). The statistical results were shown as the mean ± standard error (SE). The mean data scores were compared using Friedman’s test, complemented by a *t*-test at the 5% probability level.

## Results

### Survey of CFW Damage and Pathogen Isolate Collection

**FIGURE 1:**
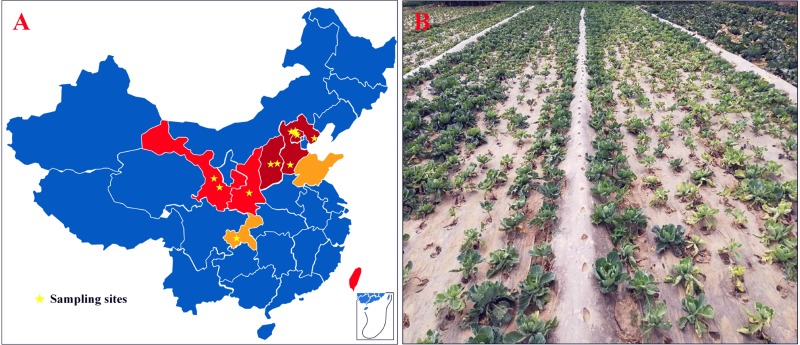
CFW threatened areas, the sampling site, and the diseased field in China. **(A)** CFW threatened areas and the sampling site; dark red areas represent damaged provinces/cities during 2001–2006; red represents areas threatened during 2007–2012; orange represents areas threatened during 2013–2017; pentagram marks the location of diseased fields. **(B)** Diseased cabbage plants in a field in Gansu Province, China.

From 2009 to 2018, surveys were conducted in cabbage-growing areas of China. The map showed that CFW was mostly distributed in the north, where the cabbage production area accounts for about 30% of the total, i.e., 900,000 ha for the whole country (Figure 1A). In 2001, CFW was first discovered in Yanqing County, Beijing (Li et al., 2003). During 2001–2006, CFW was found in the provinces around Beijing, including Hebei and Shanxi, which resulted in a production loss of about 25% for 10,000 ha (Zhang et al., 2007). During 2007–2012, CFW quickly expanded to western provinces, including Shanxi and Gansu and the southern province of Taiwan, threatening about 20% of the total planting area in the north and bringing about 35% of the production loss. From 2013 to 2018, CFW was further found in Shandong and Chongqing. To date, CFW has been found in most northern summer and autumn cabbage-growing areas and in some southern winter cabbage-growing areas, threatening approximately 30% of the summer and autumn cabbage.

According to the infection order and threat levels of CFW, three regions from Beijing and Hebei provinces were selected, respectively, where CFW was prior and most severe; two regions from Shanxi and Gansu provinces and one region from Shaanxi and Chongqing provinces were selected, respectively, for diseased plant samples collection. Finally, 12 regions were selected in six provinces of China (Figure 1A, pentagram marking). Diseased plants showed typical CFW symptoms, including retarded growth, stunting, wilting of the foliage, yellowing, and dropping of the leaves (Figure 1B). In each region, diseased plants were collected from one to four diseased fields, respectively. In total, disease plant samples were collected in 20 fields, including from 4 fields in Yanqing, Beijing, where the disease was most serious, and one field in Hechuan, Chongqing, where CFW occurred later (Table 1). Finally, 30 isolates with morphological characteristics similar to those of *F. oxysporum* were obtained from diseased plants with typical symptoms (two strains were isolated from same fields). In the pathogenicity test, all of the isolates were pathogenic to cabbage accession 01-20 and could be recovered from the inoculated and diseased plants again. Additionally, the recovered isolates had similar morphological characteristics; thus, Koch’s postulates were fulfilled, and we confirmed that the isolations were the pathogen resulting in CFW.

These strains were subjected to microscopic examination, which revealed that the hyphae were filamentous, colorless, and septate, with a width of 1.2–4.1 μm. The microconidia were colorless; hyaline; oval-ellipsoid to cylindrical, erect, or slightly curved; and mostly single-celled with a size of 1.4–5.1 × 5.2–11.8 μm. The macroconidia were sickle- or worm-shaped with one end slightly curved and colorless, consisting of three to four septa with a size of 3.2–8.6 × 32–40.8 μm. These characteristics agree with the description of FOC (Snyder and Hansen, 1940; Leslie and Summerell, 2006). Additionally, ITS and EF-1α gene sequences showed above 99% nucleotide sequence identity with FO strains (GenBank Accession Nos. KX421425 and KP964880, respectively).

Based on the above characteristics and pathogenicity survey results, these strains were identified as FOC (Booth, 1971). By removing the repeated strains from the same diseased fields, 20 isolates from 20 CFW affected fields were selected for subsequent studies finally (Table 1).

### Race Tests and Pathogenicity for the FOC Isolates

**FIGURE 2:**
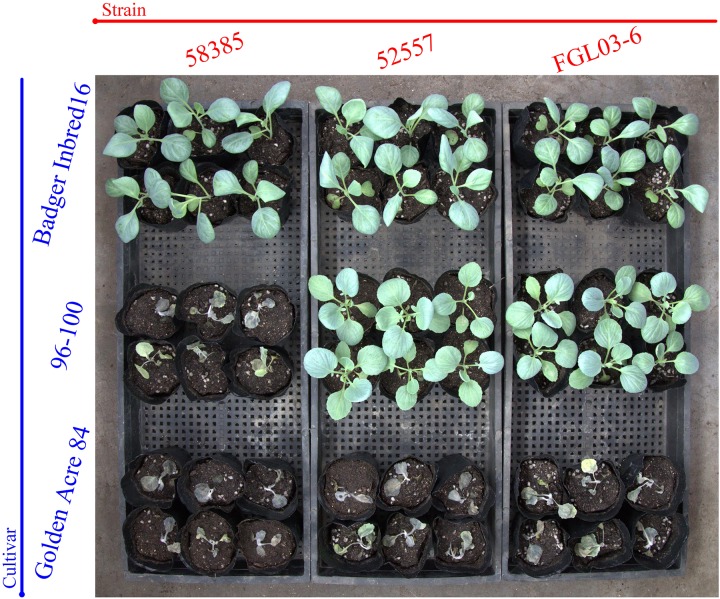
The results of race tests using cabbage cultivars for the different *Fusarium oxysporum* f. sp. *Conglutinans* (FOC) isolates. Cultivar: “Badger Inbred 16” was resistant (R) to both FOC race 1 and 2; “96-100” was R to race 1, but susceptible (S) to race 2; “Golden Acre 84” was S to both race 1 and 2 strains. Strain: FGL03-6 was recovered from diseased cabbage plants in Yanqing District of Beijing, China; 52557 and 58385, the type strains for race 1 and race 2 of FOC, provided by the American Type Culture Collection, were originally isolated from the diseased cabbage plants in Wisconsin and California in the United States, respectively.

The results indicated that 19 of 20 Chinese FOC strains resulted in typical symptoms of Fusarium wilt in the cultivars “Golden Acre 84,” “01-20,” and “Fast Vantage.” According to the pathogenicity results of these strains to three different hosts, we determined these 19 Chinese FOC strains to be FOC race 1 (Figure 2 and Table 3). Similar to the type race 1 strain 52557, 19 Chinese isolates were pathogenic to the cultivar “Golden Acre 84” and nonpathogenic to the cultivars “Badger Inbred16” and “96-100.” While the type race 2 strain 58385 was pathogenic to the cultivar “96-100.” Furthermore, the pathogenicity of the 19 Chinese strains was similar to the pathogenicity of 52557 to the cultivars “01-20” and “Fast Vantage.” In addition, there is a special strain, FOCQ, the only strain isolated from southern provinces of China, that was found to be less pathogenic to all the cultivars (Table 3).

**TABLE 3:** Disease severity of different cabbage cultivars inoculated with 25 *Fusarium oxysporum* f. sp. *conglutinans* (FOC) isolates.

**Isolate No.**	**Disease index (DI)^a^**					**Race type**
	**Badger Inbred 16**	**Golden Acre 84**	**96-100**	**01-20**	**Fast Vantage**	
FGL03-6	0.0 ± 0.0a^b^	78.7 ± 3.2abcd^c^	0.0 ± 0.0b	79.6 ± 4.2b	77.8 ± 4.8bcd	1
FOYQ-2	0.0 ± 0.0a	75.0 ± 2.8bcdef	0.0 ± 0.0b	76.9 ± 3.2bcd	75.0 ± 2.8cde	1
FOYQ-3	0.0 ± 0.0a	81.5 ± 1.6abc	0.0 ± 0.0b	82.4 ± 1.6b	80.6 ± 2.8bcd	1
FOYQ-4	0.0 ± 0.0a	74.1 ± 1.6cdefg	0.0 ± 0.0b	78.7 ± 1.6b	78.7 ± 4.2bcd	1
FOSN	0.0 ± 0.0a	79.6 ± 1.6abcd	0.0 ± 0.0b	79.6 ± 6.4b	78.7 ± 3.2bcd	1
FOCP-1	0.0 ± 0.0a	82.4 ± 5.8ab	0.0 ± 0.0b	83.3 ± 4.8b	83.3 ± 5.6ab	1
FOCP-2	0.0 ± 0.0a	76.9 ± 3.2abcde	0.0 ± 0.0b	77.8 ± 4.8bc	79.6 ± 7.0bcd	1
FOHL	0.0 ± 0.0a	70.4 ± 1.6efg	0.0 ± 0.0b	78.7 ± 5.8b	77.8 ± 2.8bcd	1
FOXT	0.0 ± 0.0a	74.1 ± 1.6cdefg	0.0 ± 0.0b	79.6 ± 1.6b	76.9 ± 1.6bcd	1
FOLT	0.0 ± 0.0a	75.9 ± 5.8bcde	0.0 ± 0.0b	80.6 ± 7.3b	77.8 ± 2.8bcd	1
FOSY-1	0.0 ± 0.0a	81.5 ± 5.8abc	0.0 ± 0.0b	82.4 ± 4.2b	79.6 ± 3.2bcd	1
FOSY-2	0.0 ± 0.0a	74.1 ± 4.2cdefg	0.0 ± 0.0b	79.6 ± 3.2b	78.7 ± 1.6bcd	1
FOPT	0.0 ± 0.0a	67.6 ± 4.2fg	0.0 ± 0.0b	71.3 ± 1.6cd	69.4 ± 2.8ef	1
FOTY-1	0.0 ± 0.0a	81.5 ± 1.6abc	0.0 ± 0.0b	83.3 ± 2.8b	81.5 ± 4.2bc	1
FOTY-2	0.0 ± 0.0a	75.9 ± 3.2bcde	0.0 ± 0.0b	80.6 ± 2.8b	77.8 ± 5.6bcd	1
FOLZ-1	0.0 ± 0.0a	77.8 ± 4.8abcde	0.0 ± 0.0b	81.5 ± 5.8b	76.9 ± 4.2bcd	1
FOLZ-2	0.0 ± 0.0a	72.2 ± 4.8defg	0.0 ± 0.0b	83.3 ± 4.8b	74.1 ± 6.4cde	1
FODX	0.0 ± 0.0a	75.0 ± 7.3bcdef	0.0 ± 0.0b	81.5 ± 1.6b	80.6 ± 0.0bcd	1
FOJY	0.0 ± 0.0a	66.7 ± 2.8g	0.0 ± 0.0b	70.4 ± 1.6d	63.9 ± 4.8f	1
FOCQ	0.0 ± 0.0a	4.6 ± 1.6h	0.0 ± 0.0b	7.4 ± 1.6e	5.6 ± 0.0g	–
52557	0.0 ± 0.0a	66.7 ± 4.8g	0.0 ± 0.0b	70.4 ± 4.2d	68.5 ± 1.6ef	1
58385	0.0 ± 0.0a	84.3 ± 1.6a	75.9 ± 4.2a	93.5 ± 1.6a	88.9 ± 2.8a	2
CS20	0.0 ± 0.0a	0.0 ± 0.0h	0.0 ± 0.0b	0.0 ± 0.0e	0.0 ± 0.0g	–
FOCAM	0.0 ± 0.0a	75.9 ± 1.6bcde	0.0 ± 0.0b	75.9 ± 3.2bcd	73.1 ± 4.2de	1
A8	0.0 ± 0.0a	66.7 ± 7.3g	0.0 ± 0.0b	70.4 ± 4.2d	65.7 ± 4.2f	1

*${}^{a}$Disease severity observed in cabbage cultivars. ${}^{b}$Mean ± standard deviation. ${}^{c}$Data followed by the same lower case letter within a column do not differ according to Friedman’s test complemented by a *t*-test at the 5% significance level.*

Eight days after inoculation, the DI of all test isolates were compared, and we found the virulence was significantly different among these strains (Table 3). FOCP-1 was the most virulent strain on “Golden Acre 84” and “Fast Vantage” with DI of 82.4 and 83.3, and FOJY had the lowest DI of 66.7 and 63.9, respectively. For the cultivar “01-20,” FOCP-1, FOTY-1, and FOLZ-2 had similar high DI value at 83.3, while FOJY had the lowest value of 70.4. Almost all the Chinese FOC strains possessed higher pathogenicity than the type strain 52557 for the three cultivars, and the pathogenicity of all these strains to the variety “01-20” was generally higher than that to the varieties “Golden Acre 84” and “Fast Vantage,” indicating that “01-20” was more S to FOC strains.

### ITS Region and EF-1α Gene Variation Analysis

**FIGURE 3:**
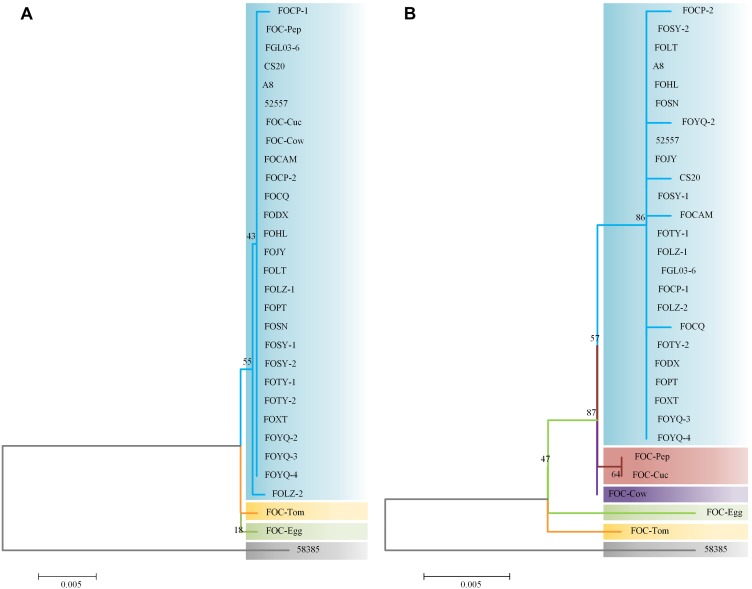
Analysis of ITS and EF-1α gene sequences using the neighbor-joining (NJ) tree method based on ITS and EF-1α gene sequences. **(A)** ITS region sequences of the 25 FOC strains and five other FO strains. **(B)** EF-1α gene sequence analysis. Branch lengths are proportional to divergence. Bootstrap frequencies from 1000 replications are noted above the branches.

Internal transcribed spacer region sequences and EF-1α gene sequences for all 25 isolates showed above 99% nucleotide sequence identity with different FO strains in NCBI (Supplementary Table S1). These ITS region and EF-1α gene sequences for the 20 FOC strains and 5 other FO *formae speciales* strains were subjected to produce a dendrogram using the NJ method with bootstrap analysis of 1000 replications. ITS region phylogenetic analysis (Figure 3A) yielded four distinctive clades, and all FOC isolates were assigned to the same clade (60% bootstrap). Some sequences from other *formae speciales* were also incorporated, including FO-Cow, FO-Cuc, and FO-Pep. The race 2 strain 58385 was in different clade and *formae speciales* FO-Egg and FO-Tom were in one clade with no other members. EF-1α gene analysis resulted in a better resolution (Figure 3B), with all FOC isolates except 58385 forming a group distinct from other *formae speciales* and having a high bootstrap support value of 86%. Interestingly, the nonpathogenic FO strain CS20 was in the same clade in both ITS region and EF-1α gene tests.

The differences between the ITS region and EF-1α gene sequence analysis indicated that the EF-1α gene was more reliable than ITS region in phylogenetic analysis. Based on ITS region sequences, FOC strains could not be distinguished from other *formae speciales*, which indicated that the ITS regions of FO strains had high homology. While EF-1α gene sequences performed better, which had richer polymorphism of *forma specialis* than ITS regions. However, the FOC race 2 strain 58385 suggests a relatively distant relationship with these FO strains whether based on the ITS region or EF-1α gene, indicating a possible divergent genome background compared with the other FOC strains and the possibility of horizontal transfer from different *formae speciales*.

### Genomic Comparisons and Evolution Status of FOC Races

There were 34.17 million paired-end reads totaling 5.13 Gb were generated by the re-sequencing of FOC race 2 58385. The assembly was 55,855,453 bp (GC content of 47.7%) in length, with an *N*_50_ value of 132,818 bp, resulting in 5,119 scaffolds. There was a total of 9,197 predicted coding genes, with an average length of 1,304 bp. The genome data has been deposited at NCBI SRA^11^ under accession no. SRX5438983.

**FIGURE 4:**
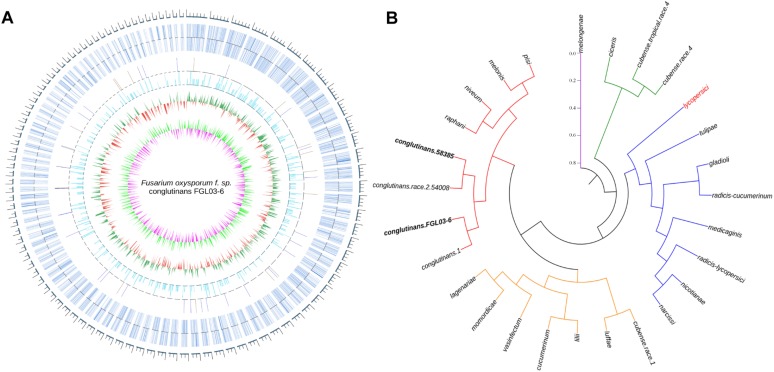
Whole-genome comparison of two FOC races and their phylogenetic analysis. **(A)** Whole-genome variations between FGL03-6 and 58385. The outermost circle in gray represents the genome scale bar; the second outermost circle in bluish gray represents gene distributions of FGL03-6; the circles in orange and blue represent insertions and deletions, respectively; the circle in light blue indicates SNPs; the circles in green and red indicate higher and lower GC-content distribution, respectively, compared with the average value; the circles in light green and purple represent higher and lower GC-skew distribution, respectively, compared with the average value. **(B)** Phylogenetic analysis of 27 FO strains from different *formae speciales* resulting in five clustering clades, using PhyML version 3.0 and iTOL software version 4.1.1 with the maximum-likelihood method.

The assembled genome of 58385 was scanned with FGL03-6 as the reference, and in total, 6,805 high-quality SNPs and 645 InDels (340 insertions and 305 deletions) were obtained. The variation type and positions between the two genomes are presented as a circular diagram in Figure 4A using Circos version 0.69. The results indicated that the genes were distributed more densely on the longer scaffolds than on the shorter ones, which might be caused by the integrity of the scaffolds. SNPs are more abundant and evenly distributed than InDels.

The two assembled genomes and 25 other *F. oxysporum* strains from 22 different *formae speciales* were aligned on the whole-genome level using the reference of *lycopersici*, which is one of the only two FO genomes that has the chromosome assembly level deposited in GenBank. In total, 2,955,397 SNPs were retrieved for the 26 genomes. A phylogenetic tree was constructed using these SNP data with PhyML version 3.0 (Guindon et al., 2010) software and iTOL software version 4.1.1 (Letunic and Bork, 2016) using the NJ method in Figure 4B. The results showed that the strains from different *formae speciales* clustered into five clades: clade I has only one member, i.e., *melongenae* (host: eggplant); clade II has *ciceris* and two *cubense* race strains; clade III has eight members with their hosts belonging to five families; clade IV has eight members, with their hosts belonging to four families; and clade V has eight members, with their hosts belonging to four families. Major parts of the *formae speciales* are clustered consistently with their hosts’ taxonomic status, such as *lycopersici* and *nicotianae* (belonging to *Solanaceae*) in clade III, *cucumerinum*, *lagenariae*, and *luffae* (belonging to *Cucurbitaceae*) in clade IV, and *conglutinans* and *raphanin* (belonging to *Cruciferae*) in clade V, indicating their close phylogenetic relationship. However, others are not, such as *narcissi* and *nicotianae* in clade III, *cucumerinum* and *lilii* in clade IV, and *melonis* and *pisi* in clade V, suggesting horizontal gene transfer between the different *formae speciales*. Specifically, the four strains of FOC are clustered to the same subclade. This hierarchical relationship is in accordance with race types, as the strains 58385 and 54008 belong to FOC race 2, while strains FGL03-6 and 1 belong to FOC race 1. The divergence of FOC races might be caused by a few significant race-specific genes. In addition, *conglutinans* also has close phylogenetic relationships with *raphanin*, *niveum*, *melonis* and *pisi*.

### Whole-Genome InDel Analysis of the FOC Strains

Based on the differential InDel loci between FGL03-6 and 58385, we designed 460 InDel primer pairs (Supplementary Table S2). After polymorphism identification, 19 InDel primer pairs (Table 4) were evenly selected as representatives of all the polymorphic primer pairs and used to perform a whole-genome analysis for all FOC isolates and the other five FO strains.

**TABLE 4:** Nineteen primer pairs used in this study for whole-genome InDel analysis of 30 *Fusarium oxysporum* isolates.

				**GC**
**Primer**			**Length**	**content**
**code**	**Direction^a^**	**Nucleotide sequence (5′–3′)**	**(nt)**	**(%)**
InDel010	F	CGTACGGTTATGATCAACAAGCA	23	43.5
	R	ATCACTACCAGAATGACTTCCCG	23	47.8
InDel073	F	ACATCGTAATTACAAAGCAAAGCT	24	33.3
	R	GGCAAAGTCTGTGTTGGAAAGTT	23	43.5
InDel090	F	GTCTGTGTCCCCTTTACCCAAG	22	54.5
	R	CGACGATGACGAGTTAACGTTTT	23	43.5
InDel128	F	CAGAGCCGATTCAGAAGCTTGT	22	50.0
	R	GACCTCCACCCTGTACTGTTTC	22	54.5
InDel136	F	AGTGCTTGTAGAGGCTCAACATG	23	47.8
	R	AATCACTAGTATCGGTGGCCAAC	23	47.8
InDel140	F	TAGCATATGTTACAGAAGCCCGG	23	47.8
	R	GCTGAAAAGCATCTGACCAGATT	23	43.5
InDel151	F	CGTAGGTTCCACGCTTCATATGT	23	47.8
	R	TGGTGAGAACTGTTCCTCGTTAG	23	47.8
InDel154	F	GTGTTACCTTCGTGCTTGGTTG	22	50.0
	R	AACATAACATCTAGGCGGTGGAG	23	47.8
InDel172	F	TTCAAGTAGTGCCAGGCTTCTC	22	50.0
	R	CTTCAGAAGAGCGGCAAAATCCA	23	47.8
InDel184	F	AGGATAGTCAATTTACAACCCCTGT	25	40.0
	R	GATCCATGTTCATTTGCAGGGTT	23	43.5
InDel206	F	GCCTTAGATATAGCGGTTCGAGT	23	47.8
	R	AAAACGATATGTGGGCTTTCGTT	23	39.1
InDel217	F	ATGAACTTGATGGGGTTACCGAT	23	43.5
	R	TTGTATTGCTGTTTGGGTGTACC	23	43.5
InDel223	F	CACACCATTCCAACGCGTTG	20	55.0
	R	TTGCGCAAAACAGGTAAAAGGAA	23	39.1
InDel254	F	CAAAGCCTGATTGCTGACGAATC	23	47.8
	R	TGTCCTGATCTAGCAAGCAAGTT	23	43.5
InDel267	F	GCGCTTGGAAAATCTTAGGATCC	23	47.8
	R	TTGTACGTGATCCAGTTGAAGGG	23	47.8
InDel311	F	GGGGTTTCTGTCACTCTCTTCAT	23	47.8
	R	TTCTCTTCCTGCAGTTCGAGAAT	23	43.5
InDel329	F	AATTCCGTACTGGCCATTTCAGA	23	43.5
	R	GATCACCTATCGGAGTTTGACAT	23	43.5
InDel410	F	AGTGACAGTTGCTTTTAGAGGCT	23	43.5
	R	TTGCCATAATGAGCAGAGTCGAT	23	43.5
InDel442	F	CAAGGGACAAGATACACCCCTAC	23	52.2
	R	GTCGCAAATGGAGAGGTCTTTCC	23	52.2

*${}^{a}$F, forward; R, reverse.*

**FIGURE 5:**
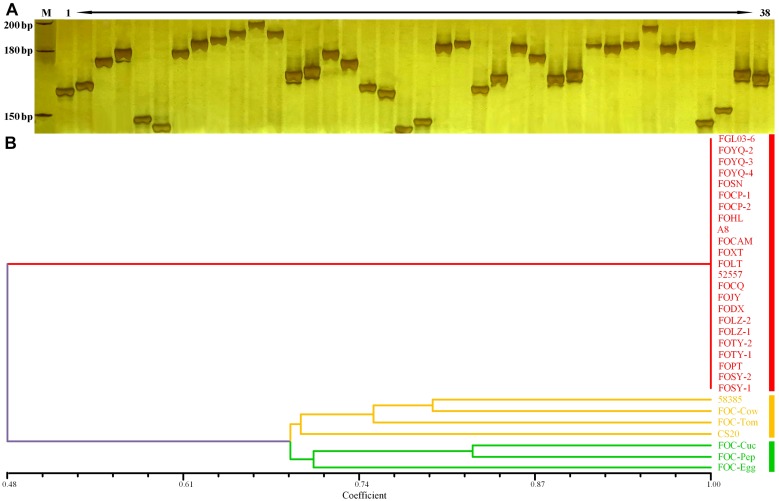
Whole-genome InDel analysis for FGL03-6 and 58385 and dendrogram generated using the whole-genome InDel polymorphism dataset for the FOC strains. **(A)** Polymorphic InDels between FGL03-6 and 58385. Lane M: DNA ladder; lanes 1–38: PCR products of polymorphic InDel primers. **(B)** Dendrogram analysis using the SAHN module of NTSYS-PC software version 2.1. The bottom scale is the percentage of Jaccard’s similarity coefficient.

The InDels polymorphism for the 19 loci was clearly visualized after the amplicons were analyzed through PAGE and silver staining (Figure 5A). The whole-genome InDel data for all isolates were analyzed with the NTSYS-PC software version 2.1 (Figure 5B). The statistical results of polymorphism of the 25 FO strains are shown in Supplementary Table S3. The results demonstrated three clades. A close relationship was displayed for all FOC race 1 strains, which all fell into the same clade I. However, the race 2 strain 58385 distinguished from FOC race 1 strains and grouped in the same clade with the *formae speciales lycopersici*, *phaseoli*, and a nonpathogenic FO strain CS20, further indicating its divergent genome background with race 1 strains, and increasing the possibility of genomic horizontal transferring from different *formae speciales*, as is indicated from the result of the EF-1α gene sequence analysis.

## Discussion

For many crops, the most effective way to control Fusarium wilt is to breed R varieties (Gordon and Okamoto, 1990; Cianchetta and Davis, 2015). Before the disease control measures are taken, the plant pathogens must be accurately detected and identified. When plants identify new virulence genes in the pathogen, the plant may evolve new defense mechanisms to limit the negative impact of the invader (Takken and Rep, 2010). *F. oxysporum* f. sp. *niveum* (E.F.Sm.) Snyder & Hansen, the causal agent of Fusarium wilt of watermelon [*Citrullus lanatus* (Thunb.) Matsum. & Nakai], is divided into four races on the basis of pathogenicity assays, and because of genetic variations, differences in virulence have been recognized and make it more difficult to control the disease (Zhou et al., 2010). Also, due to the genetic heterogeneity and the great variations of the brassica clubroot pathogen *Plasmodiophora brassicae* Woronin, the R cultivars of crucifer crops cannot achieve complete immunity to clubroot disease (Diederichsen et al., 2009). In this study, 19 Chinese FOC strains were determined to be FOC race 1 by pathogenicity tests, while these strains showed significant differences in virulence, suggesting there are higher requirements for the resistance level of cabbage cultivars in China. The inbred line “96-100” and its derived cultivars like “Zhonggan 828” and “Zhonggan 588” are HR to FOC race 1 and have been used extensively in CFW control (Zhang et al., 2014). However, these cultivars are S to FOC race 2. Although FOC race 2 has not been found in China, there might be very few resistance resources for FOC race 2 in China. Thus, FOC race 2 is undoubtedly a potential threat for cabbage resistance breeding in China, and there is a great need to discover and create more resistance resources as well as to clarify the genetic control of race 2 resistance, so as to apply in molecular resistance breeding.

Compared with the identification and classification of fungal strains by bioassays, molecular methods based on certain DNA segment polymorphisms and specific gene sequences can effectively save time and labor costs. Selecting one appropriate molecular method or taking multiple methods is also necessary, especially for the identification of virulence, *formae speciales* and subspecies (Brown and Proctor, 2013). Edel-Hermann et al. (2012) characterized the pathogens of Fusarium-diseased tomato plants in Algeria using intergenic spacer (IGS) DNA typing and PCR detection of the *SIX1* gene specific to FOL. Studies on the phylogeny of *Fusarium* spp. have shown that the translation EF-1α was a suitable genetic marker to distinguish between species (O’Donnell et al., 2004; Kristensen et al., 2005). Henry et al. (2017) identified 59 isolates of *F. oxysporum* f. sp. *fragariae* Winks & William obtained from diseased strawberry plants through pathogenicity testing and the analysis of IGS and EF-1α sequences. In this study, using ITS and EF-1α variation analysis as well as whole-genome InDel analysis, it was discovered that all Chinese FOC race 1 strains were associated with the same clade as the type race 1 strain 52557, distinguishing from the type race 2 strain 58385. In the phylogenetic analysis of this study, five other FO *formae speciales* were distinguished from FOC race 1 isolates by EF-1α variation analysis and whole-genome InDel analysis. It was indicated that the EF-1α gene sequences of Chinese FO strains showed good homology and abundance. It was effective in race identification and showed a good relationship between race and *forma specialis*. Meanwhile, the whole-genome InDel analysis will be used for *forma specialis* identification with more genetic variation locus.

With the enrichment of genomic data, more genes and DNA sequences were obtained (e.g., effectors and genomic variation information), and more reliable and rapid methods for pathogen identification and classification were developed. In this study, the re-sequencing and genome assembly of FOC race 2 strain 58385 provided a lot of mutation data for evolution and variation analysis. Analogously, Fusarium Comparative Genomics Project conducted by Ma et al. (2010) revealed interesting features concerning the *F. oxysporum* genome, including the lineage-specific (LS) chromosomes and important virulence factors. Based on the genome of *F. oxysporum* and comparing genomic analysis, Ling et al. (2016) developed a set of specific primers, which was highly specific and could be used for detecting FOC in diseased plant and infested soil. In this study, we developed genome-wide InDel markers, which could be used to study variation and evolution of *F. oxysporum* species and even races. Currently, omics methods, including high-throughput RNA sequencing (RNA-seq) and proteomics, are gaining attention. Li et al. (2015) published proteome reference maps for both FOC mycelium and conidia, identified 145 differentially expressed proteins (DEPs), and verified that one of the genes, i.e., glucanosyltransferase gene *gas1*, is essential for pathogenicity. Pu et al. (2016) investigated the xylem sap proteome of cabbage varieties with FOC-infected and identified 10 possible virulence and/or avirulence effectors. Undoubtedly, these omics methods will provide more important pathogenicity or virulence/avirulence genes and variation information for molecular characterization of the pathogens in the genomic era in the future.

In conclusion, we first reported the genetic diversity, virulence, and race test of Chinese FOC strains and showed that all the strains belonged to FOC race 1 at present, but they showed high virulence differences. Our results provided genome data and the evolutionary information of the FOC race 2 strain 58385 and discovered that whole-genome InDel variation analysis could be used for molecular identification and phylogenetic analysis of FO strains and other fungus. This work contributed to our knowledge of FOC and provided rich information for cabbage resistance breeding.

## Data Availability

All datasets generated for this study are included in the manuscript and/or the Supplementary Files. The datasets generated for this study can be found in the NCBI SRA https://www.ncbi.nlm.nih.gov/sra/SRX5438983.

## Author Contributions

XL performed the experiments and wrote the draft. MX and CK analyzed the data and prepared the figures. HL, ZF, and YY conceived the idea and designed the experiments. LY, YZ, YW, and JL revised the manuscript. All the authors have read and approved the final manuscript.

## Conflict of Interest Statement

The authors declare that the research was conducted in the absence of any commercial or financial relationships that could be construed as a potential conflict of interest.

## Footnotes

1 http://www.fao.com

2 http://genome.ucsc.edu

3 http://biobwa.sourceforge.net

4 http://samtools.sourceforge.net

5 http://circos.ca

6 http://itol.embl.de/

7 http://bio-bwa.sourceforge.net

8 http://www.htslib.org/

9 https://software.broadinstitute.org/gatk

10 http://sourceforge.net/projects/primer3/

11 https://www.ncbi.nlm.nih.gov/sra

## Supplementary Material

TABLE S1 The ITS region sequences and EF-1α gene sequences for all FO strains.

TABLE S2 InDel primer pairs for FGL03-6 and 58385.

TABLE S3 The polymorphism of 25 FO strains in 19 InDel loci.
